# The role of cytology in endobronchial ultrasound‐guided transbronchial needle aspiration: A study of 813 cases focusing on diagnostic yield, an analysis of misdiagnosed cases and diagnostic accordance rate of cytological subtyping

**DOI:** 10.1002/dc.24608

**Published:** 2020-09-07

**Authors:** Wen‐hao Ren, Shuang‐Mei Zou, Yue‐ming Zhang, Lei Zhang, Lin‐lin Zhao, Ning Lu, Jian Cao

**Affiliations:** ^1^ Department of Pathology and Resident Training Base, National Cancer Center/Cancer Hospital Chinese Academy of Medical Sciences and Peking Union Medical College Beijing China; ^2^ Department of Endoscopy, National Cancer Center/Cancer Hospital Chinese Academy of Medical Sciences and Peking Union Medical College Beijing China; ^3^ Department of Pathology, National Cancer Center/National Clinical Research Center for Cancer/Cancer Hospital and Shenzhen Hospital Chinese Academy of Medical Sciences and Peking Union Medical College Beijing China

**Keywords:** accuracy rate, cytological diagnosis, endobronchial ultrasound‐guided transbronchial needle aspiration, false‐negative, false‐positive, subtyping

## Abstract

**Background:**

Endobronchial ultrasound‐guided transbronchial needle aspiration (EBUS‐TBNA) is a minimally invasive technique for cytological and histological diagnosis. The objective of this study was to explore the role of cytological diagnosis in EBUS‐TBNAs.

**Methods:**

Eight hundred and thirteen consecutive cases performed EBUS‐TBNA with both cytological and histological diagnoses were retrospectively reviewed. All patients were followed up for clinical data.

**Results:**

Before immunohistochemical examination, the cytological sensitivity, specificity, and diagnostic accuracy of EBUS‐TBNAs were 92.9% (421/453), 98.9% (348/352), 95.5% (769/805), respectively. After immunohistochemical examination, the sensitivity, specificity, and diagnostic accuracy were 93.0% (423/455), 99.4% (348/350), 95.8% (771/805), respectively. The majority of false‐negative were cases whose cytological diagnosis was “atypical” or the cytological diagnosis suggested “inadequate.” “Neoplastic” were also prone to false‐negative cytology. The diagnostic accordance rate of cytological subtyping was 90.3% for squamous‐cell carcinoma, 99.2% for adenocarcinoma, and 98.1% for small‐cell carcinoma before immunohistochemical examination, and became 85.9%, 98.5%, and 98.2% after immunohistochemical examination, respectively.

**Conclusion:**

Cytological diagnosis in EBUS‐TBNAs had a good sensitivity and high specificity. The sensitivity and specificity of cytological diagnosis were proved to be higher after the immunohistochemical examination. At the same time, cytology had high accordance rate in subtype diagnosis. False‐negative results occurred more commonly in cases whose cytological diagnosis was “atypical” or the cytological diagnosis suggested “inadequate” or the corresponding histological diagnosis was “Neoplastic.”

**Abbreviations**
ADC: adenocarcinoma
EBUS‐TBNA: endobronchial ultrasound‐guided transbronchial needle aspiration
ICC: immunocytochemistry
IHC: immunohistochemistry
ROSE: rapid on‐site evaluation

## 1 INTRODUCTION

Endobronchial ultrasound‐guided transbronchial needle aspiration (EBUS‐TBNA) has become increasingly popular for clinical applications. EBUS‐TBNA mostly obtains samples from mediastinal and lung lesions. Because most patients rarely undergo repeated sampling and specimens are not always adequate, EBUS‐TBNA specimens are very precious, and it is of great importance to make a correct diagnosis with fewer specimens. Cytology can make use of fewer specimens to make a diagnosis; thus, it is of great clinical significance to study the value of cytology in EBUS‐TBNAs. In addition, in terms of treatment, different cancer subtypes have differential treatment options. The 2011 International Association for the Study of Lung Cancer/American Thoracic Society/European Respiratory Society Multidisciplinary Classification of Lung Adenocarcinoma mandated that pathologists subtype N‐SCLC (non‐small‐cell lung cancer) whenever feasible.^1^ Therefore, it is very important to study the role of cytological subtype diagnosis in EBUS‐TBNAs.

## 2 MATERIALS AND METHODS

### 2.1 Patients

There were 813 cases of EBUS‐TBNA with cytological and histological diagnoses from April 2014 to December 2015 at the National Cancer Center/Cancer Hospital, Chinese Academy of Medical Sciences and Peking Union Medical College. A total of 479 men (58.9%) and 334 women (41.1%) comprised the 813 patients. The median patient age was 59 years old (age range, 17‐83 years old).

### 2.2 Ethics approval and consent to participate

The Institutional Review Board of Chinese Academy of Medical Sciences and Peking Union Medical College approved this study and waived the requirement for informed consent.

### 2.3 Procedures

EBUS‐TBNA was carried out in patients under local anesthesia and performed with an echobronchoscope (BF‐UC160F‐OL8, Olympus, Tokyo, Japan). The locations, adjacent structures, and sizes of the lesions were assessed by color Doppler imaging (EU‐C2000). A dedicated aspiration needle (22‐gauge NA‐201SX‐4022) was then placed in the working channel and advanced into the lesion, the stylet was withdrawn, suction was applied to the needle, and the needle was then moved forward and backward within the lesion. A total of 10 to 15 passes per lesion was obtained. The aspirate material was placed onto a glass slide. First, the strip‐shaped component was picked from the glass and fixed in formalin, delivered to the pathology department and stained with H&E for further histological diagnosis. Then, the remainder of the aspirates on glass were smeared and immediately fixed in 95% ethanol, while the remaining aspirates obtained from flushing the needle with a small amount of saline was injected into vials of Cytolyt solution (Cytyc Company Products). Next, the smears and material vials were delivered to the cytology room. The smears were stained with H&E, while the material vials were vibrated, centrifuged, and transferred into a vial containing a preservative fluid. Then, the material vials were inserted into Thinprep 5000 for preparation, and staining was performed with Pap staining.

In some cases, immunohistochemistry (IHC) or immunocytochemistry (ICC) was performed to obtain additional information. Immunohistochemical or immunocytochemical analysis in our study was performed on an autostainer, which was a Ventana Benchmark XT (Ventana Medical Systems, Inc), according to the manufacturer's protocols. Our institution used a panel of thyroid transcription factor‐1(TTF‐1), Napsin‐A, p40, and p63 to subtype most N‐SCLCs. Synaptophysin (Syn), Chromogranin A (CgA), CD56, and Ki‐67 were used to identify small‐cell carcinomas. Other antibodies were also used when needed for tumor origin identification and differentiation between benign and malignant status. In our series, IHC was used for 125 cases of the histological samples and ICC was used for 21 cases of the cytological samples, no patient had both immunohistochemical and immunocytochemical examination.

The cytological diagnosis was based on a combination of 2 to 4 conventional smears and a liquid‐based preparation. The histological diagnosis was based on 1 to 3 H&E slides of biopsy tissue and/or surgical samples.

In the retrospective analysis, we used a six‐tiered system to reclassify the samples as follows: “nondiagnostic”; “negative for malignancy/neoplasia”; “atypical”; “neoplastic”; “suspicious (of malignancy)”; and “malignant.” “Nondiagnostic” specimen was defined as that with a cytological diagnosis of “negative for malignancy/neoplasia” and a nucleated cell quantity of less than 50 in every slide, regardless of conventional smear or liquid‐based preparation. Specimens “negative for malignancy/neoplasia” contained normal, inflammatory, or granulomatous lesions. An “atypical” cytologic specimen demonstrates cytologic features of greater dysmorphology than those assigned to the “negative for malignancy/neoplasia” category but falling short of those assigned to the suspicious for malignancy or neoplasm. Specimens that were “neoplastic” meant that the specimens were benign neoplasm, or low‐grade cancer, and the nuclear atypia were mild. In our study, these tumors included carcinoids, atypical carcinoids, and low‐grade malignant tumors of the salivary gland. The classification of “suspicious” meant that the specimen was deemed suspicious of malignancy. A “malignant” specimen indicated a malignant tumor, and malignant tumors could be further divided into “squamous‐cell carcinoma,” “adenocarcinoma,” “small‐cell carcinoma,” “non‐small‐cell carcinoma,” “unclassified carcinoma,” or “other special type.”

In addition to these six categories, when “negative for malignancy/neoplasia” specimens contained a nucleated cell quantity of more than 50 but less than 200 or “atypical” specimens contained a nucleated cell quantity of less than 200 in conventional smear or liquid‐based preparation, the cytological diagnosis will suggest the specimens were “inadequate.”

The histological and cytological diagnoses of all cases were performed by senior pathologists and cytologists.

### 2.4 Statistical analysis

All patients were followed for at least 3 years. Nondiagnostic cases were excluded from subsequent analysis, nondiagnostic rate is the number of “nondiagnostic” divided by the total number of specimens.

The gold standard for true diagnosis is based on a histological diagnosis or clinical diagnosis, and we compared the accuracy of cytological diagnosis with this gold standard.

A cytological or histological positive was defined as a diagnosis of “neoplastic,” “suspicious,” or “malignant.” A clinical positive was defined by X‐ray or CT scans showing clear lesions or metastases (including in the lungs, mediastinum, brain, liver, bone marrow, etc.) and the implementation of chemotherapy and/or radiotherapy. A cytological or histological negative was defined as a diagnosis of “negative for malignancy/neoplasia” or “atypical.” A clinical negative was defined by no growth upon repeat imaging or testing negative for malignancy upon repeat biopsy or surgery during the follow‐up period.

In our study of the cytological positive and histological negative patients, 33 had a subsequent clinical positive result. The 33 histological diagnoses were all based on biopsy tissues, and all reports noted that the tissue amounts present were minimal. The 33 cases were included as cytological true positives.

The diagnostic sensitivity, specificity, and accuracy were calculated using the following standard definitions: sensitivity—the probability of obtaining a positive test result for a subject with the disease; specificity—the probability of obtaining a negative test result for a subject without the disease; and diagnostic accuracy—the proportion of correctly classified subjects among all subjects.

In the diagnostic accordance rate of cytological subtyping for adenocarcinoma, squamous‐cell carcinoma, and small‐cell carcinoma, those cannot get a definitive subtyping were also included in the accordant cases. For example, the discordant cases of cytological adenocarcinomas were the cases whose cytological diagnosis was adenocarcinoma while the corresponding histological diagnosis was “negative for malignancy/neoplasia,” “atypical,” “other special type,” “squamous‐cell carcinoma,” or “small‐cell carcinoma”; the accordance number of cytological adenocarcinomas was the total number of cytological adenocarcinomas minus the discordant cases of cytological adenocarcinomas. The diagnostic accordance rate of cytological subtyping for adenocarcinoma was the accordance number of cytological adenocarcinomas divided by the total number of cytological adenocarcinomas.

## 3 RESULTS

### 3.1 Nondiagnostic rate and inadequate rate

Eight out of 813 cases with few cells were categorized as “nondiagnostic,” with the nondiagnostic rate of 0.98% (8/813), and the remaining 805 cases were included in the further analysis. Thirty cases suggested “inadequate,” with the inadequate rate of 3.7% (30/813).

### 3.2 Location

Of 805 EBUS‐TBNA samples, those from the peritracheal lymph node/lump accounted for 94.0%, those from the mediastinal lump/lymph nodes accounted for 2.8%, and those from the peritracheal lung lump accounted for 3.2%.

### 3.3 The diagnostic results

The diagnostic details are shown in Table 1. The numbers in brackets are the results obtained before immunohistochemical examination. IHC was carried out in 125 histological cases, and 30 cases corresponded to a changed diagnostic grade after IHC, with the specific changes shown in Table 2. ICC was carried out in 21 cytological specimens (no cases were examined by both IHC and ICC), all 21 cytological specimens were “malignant” before ICC and the purpose of the examination was to further subtype, these diagnoses grades did not change after immunocytochemical examination.

**TABLE 1:** Cytological, histological, and clinical diagnoses in EBUS‐TBNAs

Cytological diagnosis (EBUS‐TBNA)	Histological diagnosis (EBUS‐TBNA)					Clinical‐positive	Total
	Negative	Atypical	Suspicious	Neoplastic	Malignant		
Negative	325 (324)	10	4 (9)	1 (0)	16 (13)	0	356
Atypical	12 (13)	1	1	1 (0)	9	0	24
Suspicious	1	1	6 (11)	0	40 (35)	9	57
Malignant	0	0 (2)	21 (38)	1 (0)	322 (304)	24	368
Total	338	12 (14)	32 (59)	3 (0)	387 (361)	33	805

*Note*: The numbers in brackets are the results before immunohistochemical examination.

**TABLE 2:** The distribution of diagnostic grade changes before and after immunohistochemistry (IHC)

	Before IHC	After IHC
Cytological diagnosis (N)	Histological diagnosis (N)	Histological diagnosis (N)
Negative (5)	Suspicious (5)	**Negative (1)**
		Neoplastic (1)
		Malignant (3)
Atypical (1)	Negative (1)	**Neoplastic (1)**
Suspicious (5)	Suspicious (5)	Malignant (5)
Malignant (19)	Atypical (2)	**Malignant (2)**
	Suspicious (17)	Malignant (16)
		Neoplastic (1)

*Note*: N is the number of cases. After immunohistochemical verification, the qualitative diagnosis of four histological specimens (shown in bold) changed; the number of false‐negative cytology did not change(false‐negative cytology increased and decreased by one case respectively), while the number of false‐positive cytology was reduced by two cases.

### 3.4 The diagnostic yield of cytology

According to different diagnostic grades, we divided cytological diagnosis and histological/clinical diagnosis into negative and positive groups. After IHC, the groups of four cases (these four cases are shown in bold in Table 2) were changed; overall, the number of false‐negative cytology did not change (false‐negative cytology increased and decreased by one case, respectively), while the number of false‐positive cytology was reduced by two cases; this change resulted in the total number has changed from 453 to 455 for the calculation of sensitivity, and from 352 to 350 for the calculation of specificity after IHC. The diagnostic grouping results are shown in Table 3. Before immunohistochemical examination, the sensitivity, specificity, and diagnostic accuracy of cytology were 92.9% (421/453), 98.9% (348/352), and 95.5% (769/805), respectively. After immunohistochemical examination, the sensitivity, specificity, and diagnostic accuracy were 93.0% (423/455), 99.4% (348/350), and 95.8% (771/805), respectively. The sensitivity, specificity, and diagnostic accuracy of cytology all increased after immunohistochemical examination

**TABLE 3:** Cytological and histological/clinical diagnostic grouping results in EBUS‐TBNAs

Cytological and histological/clinical interpretation (EBUS‐TBNA)			
Cytological interpretation	Histological/clinical interpretation		
	Negative	Positive	Total
Negative	348	32	380
Positive	2 (4)	423 (421)	425
Total	350 (352)	455 (453)	805

*Note*: The numbers in brackets are the results before immunohistochemical examination.

### 3.5 The diagnostic results of cytology in false‐negative cases

A total of 32 (22 “negative for malignancy/neoplasia” and 10 “atypical”) cytological specimens were false negatives, 18.2% (4/22) “negative for malignancy/neoplasia” and 60% (6/10) “atypical” cases suggested “inadequate.” Cases confirmed as “neoplastic” by histopathology account for 9.1% (2/22) of “negative for malignancy/neoplasia” and 20.0% (2/10) of “atypical.” The distribution of false‐negative cases is shown in Table 4, “inadequate” accounted for 31.3% of all false negatives, and “neoplastic” accounted for 12.5%.

**TABLE 4:** The distribution of false‐negative cytological specimens

False‐negative cases	Total	Inadequate		Adequate but did not find the positive cells		The histological diagnosis was “neoplastic”	
		N	% (N/total)	N	% (N/total)	N	% (N/total)
Cytological‐negative for malignancy/neoplasia	22	4	18.2% (4/22)	18	81.8% (18/22)	2	9.1% (2/22)
Cytological‐atypical	10	6	60.0% (6/10)	4	40.0% (4/10)	2	20.0% (2/10)
Total	32	10	31.3% (10/32)	22	68.7% (22/32)	4	12.5% (4/32)

### 3.6 The diagnostic results of cytology in false‐positive cases

There were two false‐positive cases. One was diagnosed as “suspected adenocarcinoma” cytologically, while the histological diagnosis was “granulomatous nodule,” and the other was diagnosed “suspected small‐cell carcinoma,” but the histological diagnosis was “degenerative cells, not enough to diagnose malignancy.”

### 3.7 The diagnostic accordance rate of cytological subtyping

The cytological subtypes were classified into adenocarcinoma, squamous‐cell carcinoma, small‐cell carcinoma, non‐small‐cell carcinoma, other special type, and unclassified carcinoma. ICC was performed in 21 cytological cases. Before and after ICC, the total number of each subtype changed. The changes were as follows: (a) 4 cases of cytological non‐small‐cell carcinoma were further diagnosed as 2 cytological‐adenocarcinomas and 2 cytological‐squamous‐cell carcinomas; (b) 6 cases of cytological‐unclassified tumor were further diagnosed as cytological‐small‐cell carcinoma; and (c) the other 11 cases were confirmed by ICC without changes of the original subtyping. After immunocytochemical examination, the classification of squamous‐cell carcinoma, adenocarcinoma, and small‐cell carcinoma diagnosed by cytomorphlogy did not change. The subtypes of cytologically positive specimens and the corresponding histological/clinical interpretation results of the EBUS‐TBNA samples are shown in Table 5. The misclassified cases before and after IHC are shown in Table 6. The diagnostic accordance rate of cytological subtyping was 90.3% for squamous‐cell carcinoma, 99.2% for adenocarcinoma, and 98.1% for small‐cell carcinoma before immunohistochemical examination, which became 85.9%, 98.5%, and 98.2% after immunohistochemical examination, respectively (Table 7).

**TABLE 5:** The subtypes of cytologically positive specimens and corresponding histological/clinical interpretation results in EBUS‐TBNA

Cytological subtyping in EBUS‐TBNA	Histological subtyping in EBUS‐TBNA							Clinical positive	Total
	Negative or atypical	ADC	SqCC	SCC	N‐SCC	Unclassified carcinoma	Other special type		
ADC	1	110 (97)	1 (0)	0	9 (14)	6 (13)	0	6	133 (131)
SqCC	0	8 (6)	40 (37)	0	10 (12)	2 (4)	1 (0)	3	64 (62)
SCC	1 (2)	1 (0)	0	89 (74)	0	15 (24)	0	3	109 (103)
N‐SCC	0	27 (20)	2 (0)	1 (0)	4 (11)	2 (9)	0	8	44 (48)
Unclassified carcinoma	0 (1)	20 (17)	4 (3)	10 (7)	7	10 (27)	7 (2)	12	70 (76)
Other special type	0	0	0	0	0	0	4 (4)	1	5
Total	2 (4)	164 (140)	47 (40)	100 (81)	30 (44)	35 (77)	14 (6)	33	425

*Note*: The numbers in brackets are the results before immunohistochemical examination.

Abbreviations: ADC, adenocarcinoma; SqCC, Squamous cell carcinoma; SCC, small‐cell carcinoma; N‐SCC: non‐small‐cell carcinoma;

**TABLE 6:** The misclassified cases before and after immunohistochemistry (IHC)

Before IHC		After IHC	
Cytologic (N)	Histologic (N)	Cytologic (N)	Histologic (N)
Adenocarcinoma (1)	Granuloma (1)	Adenocarcinoma (2)	Granuloma (1)
			Squamous‐cell carcinoma (1)
Squamous‐cell carcinoma (6)	Adenocarcinoma (6)	Squamous‐cell carcinoma (9)	Adenocarcinoma (8)
			Neoplastic (1)
Small‐cell carcinoma (2)	Atypical (2)	Small‐cell carcinoma (2)	Atypical (1)
			Adenocarcinoma (1)

*Note*: N is the number of cases.

**TABLE 7:** The diagnostic accordant rate of cytological subtyping

	Before immunohistochemical examination	After immunohistochemical examination
ADC	99.2% (130/131)	98.5% (131/133)
SqCC	90.3% (56/62)	85.9% (55/64)
SCC	98.1% (101/103)	98.2% (107/109)

Abbreviations: ADC, adenocarcinoma; SCC, small‐cell carcinoma; SqCC, squamous‐cell carcinoma.

## 4 DISCUSSION

### 4.1 Sensitivity, specificity, and diagnostic accuracy

Standardized terminology and nomenclature for EBUS‐TBNAs are not well applied. The Papanicolaou Society of Cytopathology (P.S.C.) has developed a set of guidelines for pancreatobiliary cytology including indications for endoscopic ultrasound (EUS)‐guided fine‐needle aspiration (FNA) biopsy, techniques of EUS‐FNA, terminology and nomenclature of pancreatobiliary disease, ancillary testing, and postbiopsy treatment and management. In this classification system, EUS‐FNA specimens include six diagnostic categories: “Non‐diagnostic,” “Negative for malignancy/neoplasia,” “Atypical,” “Neoplastic (benign or other),” “Suspicious,” and “Positive/malignant,”^2^ while the P.S.C also issued guidelines on standardized terminology and nomenclature for respiratory cytology, this system include the categories “nondiagnostic,” “negative (for malignancy),” “atypical,” “neoplasm, benign neoplasm, and low‐grade malignancy,” “suspicious for malignancy,” and “malignant.”^3^ Referring to this classification systems, we used a six‐tiered system to reclassify the EBUS‐TBNA specimens as follows: “nondiagnostic,” “negative for malignancy/neoplasia,” “atypical,” “neoplastic,” “suspicious (of malignancy),” and “malignant.” The nondiagnostic and adequacy criteria for EBUS‐TBNAs were not well established,^4^ and the criteria used by various groups in the literature ranged from simply noting the presence of lymphocytes/lymphoid tissue^5^, ^6^ to more quantitative measures, such as the existence of >40 lymphocytes per high‐power field^7^ or >5 low‐power fields with >100 lymphocytes in each and <2 bronchial cell groups per low‐power field.^8^ In our study, specimens whose cytological diagnosis was “negative for malignancy/neoplasia” and which contained a nucleated cell quantity of less than 50 on the slides, regardless of conventional smear or liquid‐based preparation, were considered nondiagnostic specimens and were excluded from subsequent analysis. Our nondiagnostic rate was 0.98%, which was lower than that reported in the literature.^9^, ^10^This criterion was easier to apply in clinical diagnosis, but a minimum nucleated cell quantity of 50 as a diagnostic sample was still low, it will cause a certain false‐negative rate.

EBUS‐TBNA has been reported to have a cumulative sensitivity of 88.9% to 91.5%,^7^, ^11^, ^12^, ^13^ a cumulative specificity of 96.4% to 100%,^7^, ^11^, ^12^, ^13^ and a cumulative diagnostic accuracy of 93.0% to 93.2%,^7^, ^11^ but the sample sizes were all small. In this study, we analyzed 805 EBUS‐TBNA samples, and the diagnostic details are shown in Table 1. We calculated the diagnostic yield of cytology before and after IHC. The sensitivity, specificity, and diagnostic accuracy of the EBUS‐TBNA cytology were 93.0% (92.9%), 99.4% (98.9%), and 95.8% (95.5%) (Table 3), respectively (the percentages in brackets are the sensitivity, specificity, and diagnostic accuracy before IHC). We found that the sensitivity, specificity, and diagnostic accuracy of EBUS‐TBNA cytology all increased after IHC verification, this was because of some cytological results that were inconsistent with the histology before IHC but were confirmed to be correct after IHC verification (Table 2). To some extent, this finding can demonstrate that cytological diagnosis based on EBUS‐TBNAs has certain advantages when there are no conditions for IHC.

It is worth noting that 33 cases had a “cytological positive” but “histological negative” diagnosis. In the subsequent clinical follow‐up, these 33 cases were all verified to be clinically positive. This indicates that histological diagnoses based on tissue biopsies can give false‐negative results. Although the cell count of the 33 cytological specimens was lower, positive diagnoses were still able to be obtained, which shows that cytology has an advantage in the diagnosis of sparsely cellular specimens.

### 4.2 False‐negative and false‐positive analysis

#### 4.2.1 False‐negative analysis

There were 32 false‐negative cases, including 22 negative cases and 10 atypical cases. The distribution of false‐negative cases is shown in Table 4. One reason for the false negatives was sampling. Of the false‐negative cases, the “inadequate” accounted for 31.3% (10/32). The possible causes of inadequate specimens were as follows: (a) the locations of some samples were often deeply seated, increasing the difficulty of the operation; (b) it is difficult to obtain satisfactory samples from lesions containing large amounts of fibrotic/hyalinized/necrosis; and (c) the doctor performing the puncture did not pay enough attention to cytology and gave most of the puncture specimen to histology. In fact, in clinical practice, we found that it is better to give more specimens to cytology than to histology, especially when there are few specimens, because cytology has a higher utilization rate for fewer specimens, and it is easier to obtain a correct diagnosis when the number of aspirated cells is low. (d) Although rapid on‐site evaluation (ROSE) is a good method by which to ensure the adequacy of the samples,^14^, ^15^ we failed to implement ROSE in our hospital because of a lack of cytologists.

Inadequate sampling would also lead to a diagnosis of “atypical” if the locations were at the edge of the malignant tumor. For stromal elements of the tumor or necrosis, an “atypical” diagnosis was easy to obtain. False negatives caused by “atypical” accounted for 31.3% (10/32) of the EBUS‐TBNA cytology (Table 4).

Of course, some “atypical” cases were due to interpretation errors, cases with a histological diagnosis of “neoplastic” are often diagnosed as “atypical” in cytological interpretation. With the continuous applications of EBUS‐TBNA, the cytological diagnosis of EBUS‐TBNAs has become increasingly mature, false‐negative results caused by misinterpretation of common types of lung cancer are declining, and the proportion of false‐negative results caused by “neoplastic” increases accordingly. In our study, the category of “neoplastic” accounted for 12.5% (4/32) of all false‐negative cases，the four “neoplastic” cases included two cases of carcinoid, one case of atypical carcinoid and one case of low‐grade malignant tumors of the salivary gland. It is important to make a correct diagnosis of this category of tumor, it can not only reduces the false‐negative rate of cytological diagnosis, but also allow maximum clinical discretion in treatment, if these tumors are detected early, they are classified as more generic tumors to distinguish them from highly aggressive malignancies and to provide management flexibility for older patients with small tumors where the risk benefit of surgery is greater than that of conservative treatment.

Despite some difficulties, “neoplastic” is not undiagnosable in cytology. Carcinoid, for example, is generally identifiable in cytological specimens in previous literature reports.^16^, ^17^, ^18^ Carcinoid tumor cells are discohesive, fragments of capillaries are a helpful clue if present. Necrosis is absent and the background is clean. Individual cells are small and have round, oval, or spindle‐shaped nuclei. Nuclear outlines are smooth. Chromatin is finely granular and nucleoli are not present. In addition to the three types of tumors that were involved in our study, “neoplastic” also includes “pulmonary hamartoma,” “sclerosing pneumocytoma,” “myoepithelial neoplasms,” and so on. The cytological characteristics of these tumors have been described in detail in the literature.^3^ On the premise of understanding the cytological characteristics of these tumors, combined with clinical, imaging and immunocytochemical examination, it is possible to make a correct cytological diagnosis of these tumors to some extent, the key to making the right diagnosis is to be aware of the presence of such tumors.

#### 4.2.2 False‐positive cases

There were two false‐positive cases, including one with a cytological diagnosis of “suspicious malignancy” but the histological diagnosis of a granulomatous lesion. From the 805 EBUS‐TBNAs, 119 cases of granulomatous lesions were diagnosed by histology, while the cytological diagnoses included 25 “granulomatous,” 93 “negative for malignancy/neoplasia,” and 1 “suspicious malignancy.” The sensitivity of a “granulomatous” diagnosis in cytology was 21.0%, which was lower than that reported in the literature.^19^, ^20^ The other false‐positive case was a suspected cytologically small‐cell carcinoma, but the histological diagnosis was “degenerative cells, not enough to diagnose malignancy.” The follow‐up examination of this patient was not carried out in our hospital, so we still consider this patient as a false‐positive case.

### 4.3 The accordance rate of tumor subtyping in cytology

It is of great importance to subclassify tumors for clinical therapy. In this series, the diagnostic details of cytological subtyping are shown in Tables 5, 6, 7. The EBUS‐TBNA accordance rate of cytological subtyping was 85.9% (90.3%) for squamous‐cell carcinoma, 98.5% (99.2%) for adenocarcinoma, and 98.2% (98.1%) for small‐cell carcinoma (the numbers in brackets are the results before immunohistochemical examination), and this result demonstrates that cytology does have the potential to accurately subtype EBUS‐TBNAs, although some cases also produce classification errors. The main reason for misdiagnosed tumor subtyping was difficulty in distinguishing poorly differentiated squamous‐cell carcinoma, adenocarcinoma, and small‐cell carcinoma categories. In our study, eight cases of poorly differentiated adenocarcinoma were misdiagnosed as squamous‐cell carcinoma, one poorly differentiated adenocarcinoma was misdiagnosed as small‐cell carcinoma, and one case of poorly differentiated squamous‐cell carcinoma was misdiagnosed as adenocarcinoma. Morphological examinations of cytology samples are limited in the diagnosis of poorly differentiated cancer. ICC is a powerful tool with which to improve the accordance rate of cytology for subtyping.^21^ In the current study, the cases of misdiagnosed tumor subtypings did not implement ICC. For the 805 EBUS‐TBNA specimens with cytology and histology, the implementation ratio of ICC and IHC was 1:6 (21‐125). In recent years, with the continuous improvement of cell block technology, it is completely feasible to implement IHC on cell blocks.^22^, ^23^ It is believed that with continued clinical application, the role of cell blocks in subtyping can be further improved and that the diagnostic accordance rate of cytological subtyping will be further improved.

Our research included a deep discussion of the cytological value of EBUS‐TBNA, but there were some limitations in our study. First, although the data collected in this research were large in number, this single‐center study possibly included some bias. Second, ROSE was not used. Third, our study was a retrospective study, and immunohistochemical examination was not performed on all cases with undetermined subtypes, so the accordance rate of our tumor subtyping was inaccurate.

## 5 CONCLUSIONS

In summary, in this study, we investigated a large series of EBUS‐TBNA results and observed that this method has good sensitivity, high specificity, and a high accordance rate with tumor subtyping based on cytology. Cytological diagnoses of EBUS‐TBNA samples have high value in clinical applications. Cytology has a certain diagnostic advantage when there are few puncture specimens, which can make up for the occurrence of false‐negative histology results, but the existence of too few cells is also the main reason for a decline in the cytological diagnostic efficiency.

## CONFLICT OF INTEREST

The authors have no conflicts of interest relevant to this article.

## AUTHOR CONTRIBUTIONS

J.C. conceived the project idea. L.Z. and Y.‐m.Z. performed all endoscopic procedures. L.‐l.Z. performed smear/section preparation. J.C., S.‐M.Z. and N.L. were responsible for cytological and histological analysis. W.‐h.R. collected the data and conducted the analysis. All authors interpreted and discussed the results. W.‐h.R. and J.C. wrote the manuscript. All authors have read and approved the final version.

## Data Availability

The data that support the findings of this study are available from the corresponding author upon reasonable request.

## References

[1] Travis WD , Brambilla E , Noguchi M , et al. International association for the study of lung cancer/american thoracic society/european respiratory society international multidisciplinary classification of lung adenocarcinoma. J Thorac Oncol. 2011;6(2):244‐285. 21252716 10.1097/JTO.0b013e318206a221 PMC4513953

[2] Pitman MB , Centeno BA , Ali SZ , et al. Standardized terminology and nomenclature for pancreatobiliary cytology: the Papanicolaou Society of Cytopathology guidelines. Diagn Cytopathol. 2014;42(4):338‐350. 24554455 10.1002/dc.23092

[3] Layfield LJ , Baloch Z , Elsheikh T , et al. Standardized terminology and nomenclature for respiratory cytology: the Papanicolaou Society of Cytopathology guidelines. Diagn Cytopathol. 2016;44(5):399‐409. 26990836 10.1002/dc.23457

[4] Alsharif M , Andrade RS , Groth SS , Stelow EB , Pambuccian SE . Endobronchial ultrasound‐guided transbronchial fine‐needle aspiration: the University of Minnesota experience, with emphasis on usefulness, adequacy assessment, and diagnostic difficulties. Am J Clin Pathol. 2008;130(3):434‐443. 18701418 10.1309/BLLQF8KDHWW6MJNQ

[5] Yasufuku K , Pierre A , Darling G , et al. A prospective controlled trial of endobronchial ultrasound‐guided transbronchial needle aspiration compared with mediastinoscopy for mediastinal lymph node staging of lung cancer. J Thorac Cardiovasc Surg. 2011;142(6):1393‐400.e1. 21963329 10.1016/j.jtcvs.2011.08.037

[6] Rice DC , Steliga MA , Stewart J , et al. Endoscopic ultrasound‐guided fine needle aspiration for staging of malignant pleural mesothelioma. Ann Thorac Surg. 2009;88(3):862‐868. discussion 868‐9. 19699913 10.1016/j.athoracsur.2009.05.022

[7] Cameron SEH , Andrade RS , Pambuccian SE . Endobronchial ultrasound‐guided transbronchial needle aspiration cytology: a state of the art review. Cytopathology. 2010;21(1):6‐26. 20015257 10.1111/j.1365-2303.2009.00722.x

[8] Nayak A , Sugrue C , Koenig S , Wasserman PG , Hoda S , Morgenstern NJ . Endobronchial ultrasound‐guided transbronchial needle aspirate (EBUS‐TBNA): a proposal for on‐site adequacy criteria. Diagn Cytopathol. 2012;40(2):128‐137. 22246929 10.1002/dc.21517

[9] Mehta HJ , Tanner NT , Silvestri G , et al. Outcome of patients with negative and unsatisfactory cytologic specimens obtained by endobronchial ultrasound‐guided transbronchial fine‐needle aspiration of mediastinal lymph nodes. Cancer Cytopathol. 2015;123(2):92‐97. 25186645 10.1002/cncy.21482 PMC4502962

[10] Nambirajan A , Longchar M , Madan K , et al. Endobronchial ultrasound‐guided transbronchial needle aspiration cytology in patients with known or suspected extra‐pulmonary malignancies: a cytopathology‐based study. Cytopathology. 2019;30(1):82‐90. 30444548 10.1111/cyt.12656

[11] Luo GY , Cai PQ , He JH , et al. Application of endobronchial ultrasound‐guided transbronchial needle aspiration in the management of mediastinal and hilar lymphadenopathy without intrapulmonary mass: experience from the largest cancer center of southern China. Cell Biochem Biophys. 2013;67(3):1533‐1538. 23723003 10.1007/s12013-013-9657-x

[12] Vaidya PJ , Saha A , Kate AH , et al. Diagnostic value of core biopsy histology and cytology sampling of mediastinal lymph nodes using 21‐gauge EBUS‐TBNA needle. J Cancer Res Ther. 2016;12(3):1172‐1177. 28054531 10.4103/0973-1482.197535

[13] Sun W , Song K , Zervos M , et al. The diagnostic value of endobronchial ultrasound‐guided needle biopsy in lung cancer and mediastinal adenopathy. Diagn Cytopathol. 2010;38(5):337‐342. 19890836 10.1002/dc.21195

[14] Guo H , Liu S , Guo J , et al. Rapid on‐site evaluation during endobronchial ultrasound‐guided transbronchial needle aspiration for the diagnosis of hilar and mediastinal lymphadenopathy in patients with lung cancer. Cancer Lett. 2016;371(2):182‐186. 26656954 10.1016/j.canlet.2015.11.038

[15] Trisolini R , Cancellieri A , Tinelli C , et al. Rapid on‐site evaluation of transbronchial aspirates in the diagnosis of hilar and mediastinal adenopathy: a randomized trial. Chest. 2011;139(2):395‐401. 21030491 10.1378/chest.10-1521

[16] Geisinger KR , Stanley MW , Raab S , AA SJF . Modern Cytopathology. New York, NY: Churchill Livingstone; 2003.

[17] Anraku M , Pierre AF , Nakajima T , et al. Endobronchial ultrasound‐guided transbronchial needle aspiration in the management of previously treated lung cancer. Ann Thorac Surg. 2011;92(1):251‐255. discussion 255. 21592457 10.1016/j.athoracsur.2011.03.007

[18] Delattre C , Fournier C , Bouchindhomme B , et al. Endoscopic ultrasound guided transbronchial fine needle aspiration: a French department of pathology's 4‐year experience. J Clin Pathol. 2011;64(12):1117‐1122. 21965831 10.1136/jclinpath-2011-200382

[19] Chee A , Khalil M , Stather DR , MacEachern P , Field SK , Tremblay A . Cytologic assessment of endobronchial ultrasound‐guided transbronchial needle aspirates in sarcoidosis. J Bronchology Interv Pulmonol. 2012;19(1):24‐28. 23207259 10.1097/LBR.0b013e3182442925

[20] Navasakulpong A , Auger M , Gonzalez AV . Yield of EBUS‐TBNA for the diagnosis of sarcoidosis: impact of operator and cytopathologist experience. BMJ Open Respir Res. 2016;3(1):e000144. 10.1136/bmjresp-2016-000144 PMC4985919 27547408

[21] Dong Z , Li H , Jiang H , Wu C . Evaluation of cytology in lung cancer diagnosis based on EBUS‐TBNA. J Cytol. 2017;34(2):73‐77. 28469313 10.4103/0970-9371.203567 PMC5398023

[22] Lourido‐Cebreiro T , Leiro‐Fernández V , Tardio‐Baiges A , et al. The contribution of cell blocks in the diagnosis of mediastinal masses and hilar adenopathy samples from echobronchoscopy. Arch Bronconeumol. 2014;50(7):267‐271. 24439256 10.1016/j.arbres.2013.11.022

[23] Sanz‐Santos J , Serra P , Andreo F , Llatjós M , Castellà E , Monsó E . Contribution of cell blocks obtained through endobronchial ultrasound‐guided transbronchial needle aspiration to the diagnosis of lung cancer. BMC Cancer. 2012;12:34. 22264305 10.1186/1471-2407-12-34 PMC3292510

